# Supplementary material for: GAN-enhanced machine learning and metabolic modeling identify reprogramming in pancreatic cancer
Source: PLoS Comput Biol. 2026 Jan 2;22(1):e1013862. doi: 10.1371/journal.pcbi.1013862 (PMC12779136; doi:10.1371/journal.pcbi.1013862)
Supplement: S1 Fig — t-SNE plot showing the distribution of GAN-generated healthy samples (green), real healthy samples (blue), and cancer samples (red) in a reduced two-dimensional space. The GAN-generated healthy samples effectively follow the trajectory established by the original healthy samples, validating our generative approach and biological filtration process. This alignment confirms that our WGAN-GP model successfully learned the underlying distribution of healthy pancreatic tissue gene expression patterns. The cancer samples display a more dispersed, heterogeneous distribution that frequently overlaps with regions occupied by healthy samples, illustrating the inherent complexity of distinguishing between healthy and cancerous metabolic states based on gene expression alone. (PDF) [file pcbi.1013862.s001.pdf]

S1 Fig: The t-SNE plot demonstrates that the GAN-generated healthy samples (green dots) effectively follow the trajectory established by the original healthy samples (blue dots), validating our generative approach and biological filtration process. This alignment confirms that our WGAN-GP model successfully learned the underlying distribution of healthy pancreatic tissue gene expression patterns. Notably, the cancer samples (red dots) display a more dispersed, heterogeneous distribution that frequently overlaps with regions occupied by healthy samples, illustrating the inherent complexity of distinguishing between healthy and cancerous metabolic states based on gene expression alone. This pattern of partial overlap underscores the challenge of cancer classification and highlights the value of our integrated approach that combines transcriptomic data with genome-scale metabolic modeling to achieve high classification accuracy.

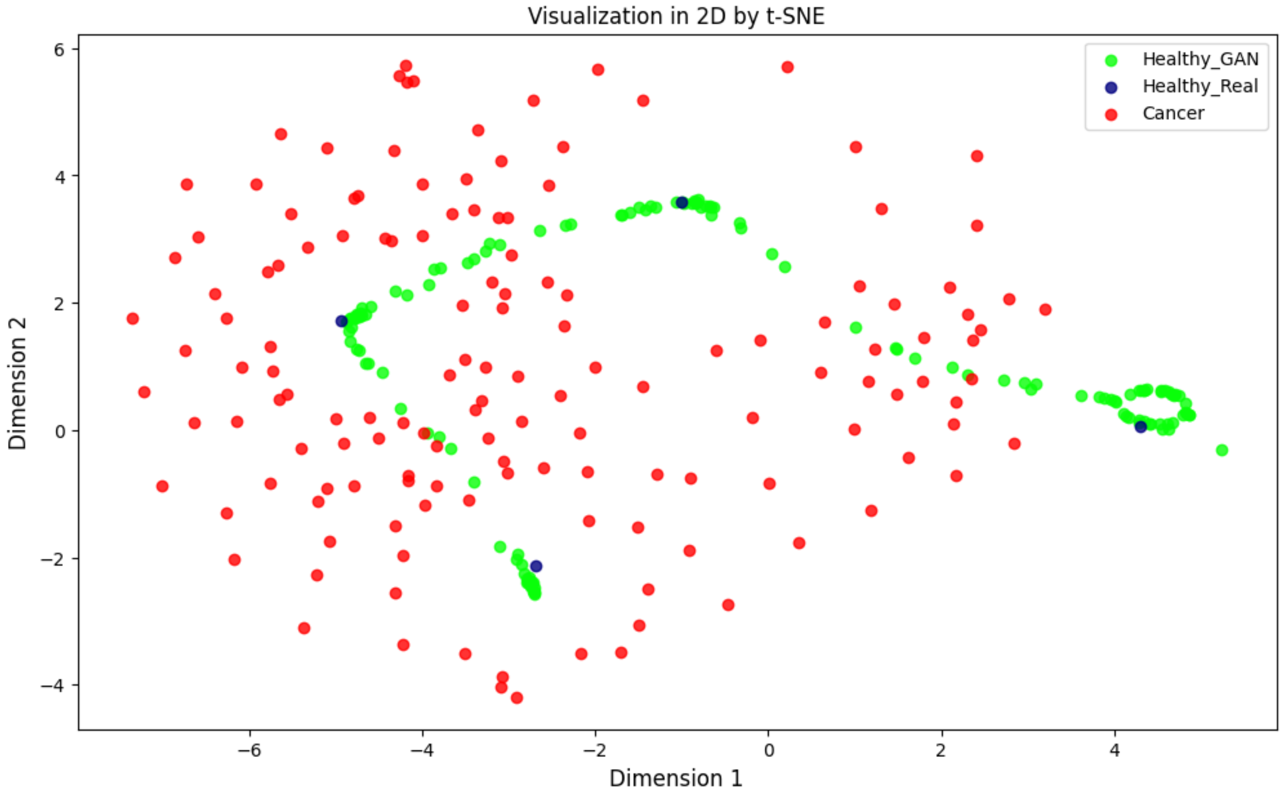

S1 Fig: t-SNE visualization of gene expression data showing the distribution of GAN-generated healthy samples (green), real healthy samples (blue), and cancer samples (red) in a reduced two-dimensional space.
