# Supplementary material for: GAN-enhanced machine learning and metabolic modeling identify reprogramming in pancreatic cancer
Source: PLoS Comput Biol. 2026 Jan 2;22(1):e1013862. doi: 10.1371/journal.pcbi.1013862 (PMC12779136; doi:10.1371/journal.pcbi.1013862)
Supplement: S2 Fig — Confusion matrix showing the classification performance of the random forest model in distinguishing between healthy and cancerous metabolic states. The model correctly identified 26 healthy and 29 cancerous cases, with only 3 healthy cases misclassified as cancerous and no false negative predictions, demonstrating high accuracy (94.83%) and perfect recall (1.0) for cancerous cases. This perfect recall for cancerous cases (29/29 correctly identified) is particularly significant in the context of cancer screening, where false negatives (missing cancer diagnoses) can have severe clinical consequences. (PDF) [file pcbi.1013862.s002.pdf]

S2 Fig: Confusion matrix showing the classification performance of the random forest model in distinguishing between healthy and cancerous metabolic states. The model correctly identified 26 healthy and 29 cancerous cases, with only 3 healthy cases misclassified as cancerous and no false negative predictions. This perfect recall for cancerous cases (29/29 correctly identified) is particularly significant in the context of cancer screening, where false negatives (missing cancer diagnoses) can have severe clinical consequences. The absence of any cancer samples being misclassified as healthy demonstrates the model's high sensitivity, a critical characteristic for potential diagnostic applications. Overall, the model achieved 94.83% accuracy while maintaining 100% sensitivity, suggesting its potential utility as a reliable screening tool that minimizes the risk of missed cancer diagnoses.

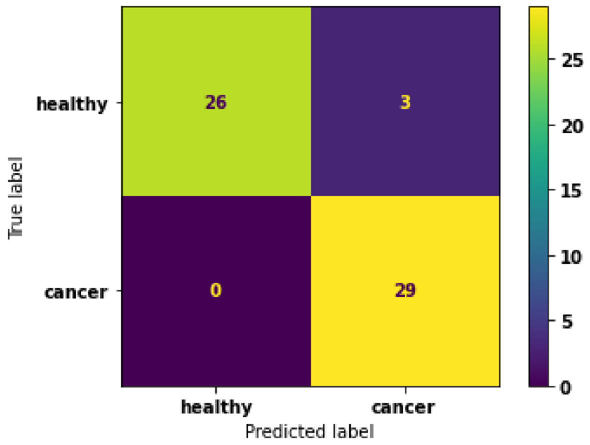

S2 Fig: Confusion matrix showing the classification performance of the random forest model in distinguishing between healthy and cancerous metabolic states. The model correctly identified 26 healthy and 29 cancerous cases, with only 3 healthy cases misclassified as cancerous and no false negative predictions, demonstrating high accuracy (94.83%) and perfect recall for cancerous cases.
