# Supplementary material for: GAN-enhanced machine learning and metabolic modeling identify reprogramming in pancreatic cancer
Source: PLoS Comput Biol. 2026 Jan 2;22(1):e1013862. doi: 10.1371/journal.pcbi.1013862 (PMC12779136; doi:10.1371/journal.pcbi.1013862)
Supplement: S1 Table — Mean flux values for top-ranked metabolic reactions comparing real healthy samples (n = 4), synthetic healthy samples (n = 140), and cancer samples (n = 144). Fold-changes are calculated as cancer flux relative to real healthy flux. The consistency of directional changes and magnitude of fold-changes between cancer and both real and synthetic healthy samples confirms that identified metabolic signatures are not artifacts of synthetic data generation. (PDF) [file pcbi.1013862.s003.pdf]

S1 Table: Validation of Key Metabolic Findings in Real Healthy Samples

Reaction/Pathway	Real-Healthy (n=4) Mean Flux	Synthetic-Healthy (n=140) Mean Flux	Cancer (n=144) Mean Flux	Fold-Change (Cancer vs Real-Healthy)
**MAR00336** (Nervonic acid transport)	0.15	0.14	6.54	+44.0×
**Heparan Sulfate Degradation**				
MAR07238	23.3	19.4	6.7	-3.5×
MAR07241	23.3	16.3	3.4	-6.9×
MAR07248	23.3	16.3	3.4	-6.9×

Table shows that cancer-associated metabolic patterns (increased nervonic acid transport, decreased heparan sulfate degradation) are consistent when comparing cancer samples to either real or synthetic healthy samples, validating that findings are not artifacts of synthetic data generation.
