# Supplementary material for: GAN-enhanced machine learning and metabolic modeling identify reprogramming in pancreatic cancer
Source: PLoS Comput Biol. 2026 Jan 2;22(1):e1013862. doi: 10.1371/journal.pcbi.1013862 (PMC12779136; doi:10.1371/journal.pcbi.1013862)
Supplement: Attachment — Submitted filename: Response to Reviewers.docx [file pcbi.1013862.s006.docx]

**Additional Editor Comments:**

comments – Red color

Our Response – Blue color

Additions to the manuscript – Black color

1) Please ensure that the CRediT author contributions listed for every co-author are completed accurately and in full.
At this stage, the following Authors/Authors require contributions: Tahereh Razmpour, Arman Roohi, Masoud Tabibian, and Rajib Saha. Please ensure that the full contributions of each author are acknowledged in the "Add/Edit/Remove Authors" section of our submission form.
The list of CRediT author contributions may be found here: [https://journals.plos.org/ploscompbiol/s/authorship#loc-author-contributions](https://urldefense.com/v3/__https:/journals.plos.org/ploscompbiol/s/authorship*loc-author-contributions__;Iw!!PvXuogZ4sRB2p-tU!A7Vk-9x6scWzsdan3qU4UhWogR5wUDyZJJMSp49GqbzYxM8N9mISyxUJgDzC3e3qd0q3KL2fmwhESHCOXnE049Sv$)

We have updated the author contributions in the submission system with complete CRediT categories for all authors as follows:

- **Tahereh Razmpour:** Conceptualization, Methodology, Software, Investigation, Data Curation, Writing - Original Draft, Visualization
- **Masoud Tabibian:** Methodology, Data Curation, Writing - Review & Editing, Visualization
- **Arman Roohi:** Methodology, Writing - Review & Editing
- **Rajib Saha:** Conceptualization, Methodology, Writing - Review & Editing, Supervision, Funding Acquisition, Project Administration

2) We have noticed that you have uploaded Supporting Information files, but you have not included a list of legends. Please add a full list of legends for your Supporting Information files after the references list.

We have added the following Supporting Information legends after the References section in the manuscript:

**Supporting Information**

**S1 Fig. t-SNE visualization of gene expression data.** t-SNE plot showing the distribution of GAN-generated healthy samples (green), real healthy samples (blue), and cancer samples (red) in a reduced two-dimensional space. The GAN-generated healthy samples effectively follow the trajectory established by the original healthy samples, validating our generative approach and biological filtration process. This alignment confirms that our WGAN-GP model successfully learned the underlying distribution of healthy pancreatic tissue gene expression patterns. The cancer samples display a more dispersed, heterogeneous distribution that frequently overlaps with regions occupied by healthy samples, illustrating the inherent complexity of distinguishing between healthy and cancerous metabolic states based on gene expression alone.

**S2 Fig. Random Forest classification performance.** Confusion matrix showing the classification performance of the random forest model in distinguishing between healthy and cancerous metabolic states. The model correctly identified 26 healthy and 29 cancerous cases, with only 3 healthy cases misclassified as cancerous and no false negative predictions, demonstrating high accuracy (94.83%) and perfect recall (1.0) for cancerous cases. This perfect recall for cancerous cases (29/29 correctly identified) is particularly significant in the context of cancer screening, where false negatives (missing cancer diagnoses) can have severe clinical consequences.

**S1 Table. Validation of key metabolic findings in real healthy samples.** Mean flux values for top-ranked metabolic reactions comparing real healthy samples (n=4), synthetic healthy samples (n=140), and cancer samples (n=144). Fold-changes are calculated as cancer flux relative to real healthy flux. The consistency of directional changes and magnitude of fold-changes between cancer and both real and synthetic healthy samples confirms that identified metabolic signatures are not artifacts of synthetic data generation.

**S2 File. Complete gene family lists.** Complete lists of all genes in each gene family mentioned in Figure 3C, organized by importance tier.

3) Some material included in your submission may be copyrighted. According to PLOSu2019s copyright policy, authors who use figures or other material (e.g., graphics, clipart, maps) from another author or copyright holder must demonstrate or obtain permission to publish this material under the Creative Commons Attribution 4.0 International (CC BY 4.0) License used by PLOS journals. Please closely review the details of PLOSu2019s copyright requirements here: PLOS Licenses and Copyright. If you need to request permissions from a copyright holder, you may use PLOS's Copyright Content Permission form.
Please respond directly to this email and provide any known details concerning your material's license terms and permissions required for reuse, even if you have not yet obtained copyright permissions or are unsure of your material's copyright compatibility. Once you have responded and addressed all other outstanding technical requirements, you may resubmit your manuscript within Editorial Manager. 
Potential Copyright Issues:
i) Figures 1, 2, and 6. Please confirm whether you drew the images / clip-art within the figure panels by hand. If you did not draw the images, please provide (a) a link to the source of the images or icons and their license / terms of use; or (b) written permission from the copyright holder to publish the images or icons under our CC BY 4.0 license. Alternatively, you may replace the images with open source alternatives. See these open source resources you may use to replace images / clip-art:
- <https://commons.wikimedia.org>
- [https://openclipart.org/](https://urldefense.com/v3/__https:/openclipart.org/__;!!PvXuogZ4sRB2p-tU!A7Vk-9x6scWzsdan3qU4UhWogR5wUDyZJJMSp49GqbzYxM8N9mISyxUJgDzC3e3qd0q3KL2fmwhESHCOXlci2Ym5$).

All images and graphics in Figures 1, 2, and 6 were created by the authors using the following tools:

- **Figure 1:** Created using Microsoft Visio and BioRender (https://biorender.com). BioRender graphics are used under an academic license agreement that permits publication under CC BY 4.0.
- **Figure 2:** Created using Python (matplotlib library) and Microsoft Visio by the authors.
- **Figure 6:** Created using Microsoft Visio by the authors.

All cell/organelle illustrations and schematic elements were either drawn by hand in Visio or created using BioRender's licensed scientific illustration library. No copyrighted third-party images were used.

4) Please amend your detailed Financial Disclosure statement. This is published with the article. It must therefore be completed in full sentences and contain the exact wording you wish to be published.
1) State the initials, alongside each funding source, of each author to receive each grant. For example: "This work was supported by the National Institutes of Health (####### to AM; ###### to CJ) and the National Science Foundation (###### to AM)."
2) State what role the funders took in the study. If the funders had no role in your study, please state: "The funders had no role in study design, data collection and analysis, decision to publish, or preparation of the manuscript."
3) If any authors received a salary from any of your funders, please state which authors and which funders..
If you did not receive any funding for this study, please simply state: u201cThe authors received no specific funding for this work.u201d

This work was supported by the National Institutes of Health (NIH MIRA Award 5R35GM143009 to R.S.). The funders had no role in study design, data collection and analysis, decision to publish, or preparation of the manuscript.

5) Thank you for stating 'All the codes and materials used for this study are available at [https://github.com/ssbio/PDAC](https://urldefense.com/v3/__https:/github.com/ssbio/PDAC__;!!PvXuogZ4sRB2p-tU!A7Vk-9x6scWzsdan3qU4UhWogR5wUDyZJJMSp49GqbzYxM8N9mISyxUJgDzC3e3qd0q3KL2fmwhESHCOXpzyJ21J$)' Please note that, though access restrictions are acceptable now, your entire minimal dataset will need to be made freely accessible if your manuscript is accepted for publication. This policy applies to all data except where public deposition would breach compliance with the protocol approved by your research ethics board. If you are unable to adhere to our open data policy, please kindly revise your statement to explain your reasoning and we will seek the editor's input on an exemption.

We confirm that all code and data at https://github.com/ssbio/PDAC are currently publicly accessible and will remain freely available without restrictions. The repository contains all computational code, processed data files, and documentation necessary to reproduce the analyses presented in this manuscript. The raw gene expression data used in this study are publicly available from The Cancer Genome Atlas (TCGA) database."

**Reviewers' comments:**

Reviewers’ comments – Red color

Our Response – Blue color

Additions to the manuscript – Black color

**Reviewer #1 comments:**

1- Biomarkers usually refer to unique metabolites or proteins. For cancer cells, the disease is due to genetic mutations. Therefore, the use of FBA to identify biomarkers is confusing to me. The flux value is determined by known multiple enzyme reactions as well as reaction thermodynamics. FBA may not have good resolution to reveal biomarker.

We thank the reviewer for this important clarification regarding terminology. We acknowledge that traditional biomarkers refer to measurable biological molecules (metabolites, proteins, nucleic acids) that can be directly quantified in clinical samples. We have revised our manuscript to more accurately describe our computational findings.

Our approach identifies metabolic signatures and therapeutic vulnerabilities rather than traditional clinical biomarkers. Specifically, we use FBA-derived flux distributions as a pattern recognition tool to systematically identify which metabolic reactions, pathways, and genes show the most significant alterations between healthy and cancerous metabolic states. The flux values themselves are not proposed as directly measurable clinical biomarkers; rather, they reveal metabolic dysfunctions that point to:

- Gene-level targets (FABPs, SLC27As, ACSLs, ACSBGs) whose expression levels could be validated experimentally as potential biomarkers
- Pathway-level dysregulation (heparan sulfate degradation, O-glycan metabolism) providing mechanistic understanding of disease pathogenesis
- Specific metabolic reactions showing altered activity that nominate processes for experimental validation

While FBA cannot directly measure metabolite concentrations, it provides complementary insights by integrating gene expression data with metabolic network constraints to predict functional consequences. Importantly, our multi-level analysis connects genetic drivers (gene expression changes) to their functional metabolic consequences, generating mechanistic hypotheses that can guide experimental biomarker validation.

We have revised the manuscript throughout to use more precise terminology: "metabolic signatures," "therapeutic targets," and "computational predictions" rather than "biomarkers" in contexts where direct clinical measurement is implied. We retain "biomarker" only when referring to the identified genes whose expression could be experimentally validated, making clear that experimental validation would be required before clinical application. The following revisions were made specifically to reflect the distinctions outlined above.

**SPECIFIC TEXT REVISIONS**

**1. TITLE**

**ORIGINAL:** "GAN-Enhanced Machine Learning and Metabolic Modeling Identify Reprogramming in Pancreatic Cancer"

**REVISED:** "GAN-Enhanced Machine Learning and Metabolic Modeling Reveal Metabolic Reprogramming and Therapeutic Targets in Pancreatic Cancer"

**2. ABSTRACT**

**ORIGINAL:** "We developed a novel computational framework integrating genome-scale metabolic modeling with machine learning to identify metabolic biomarkers and vulnerabilities in pancreatic cancer."

**REVISED:** "We developed a novel computational framework integrating genome-scale metabolic modeling with machine learning to identify metabolic signatures and therapeutic vulnerabilities in pancreatic cancer."

**ORIGINAL:** "These findings enhance our understanding of pancreatic cancer metabolism and present potential therapeutic targets..."

**REVISED:** "These findings enhance our understanding of pancreatic cancer metabolism and identify potential therapeutic targets for experimental validation..."

**3. INTRODUCTION (Multiple Locations)**

**Location 1 (Early Introduction):**

**ORIGINAL:** "The reconstruction of these cancer-specific GSMs and their subsequent constrained-based analysis aim to identify potential therapeutic targets and biomarkers across different malignancies"

**REVISED:** "The reconstruction of these cancer-specific GSMs and their subsequent constraint-based analysis aim to identify potential therapeutic targets and metabolic vulnerabilities across different malignancies"

**Location 2 (Gap Statement):**

**ORIGINAL:** "In this work, we address these gaps by developing a novel framework that combines GSM, constraint-based modeling, and ML techniques to identify PDAC-specific biomarkers and metabolic vulnerabilities."

**REVISED:** "In this work, we address these gaps by developing a novel framework that combines GSM, constraint-based modeling, and ML techniques to identify PDAC-specific metabolic signatures and therapeutic vulnerabilities."

**4. FIGURE 1 CAPTION**

**ORIGINAL:** "This integrated approach combines transcriptomics data with metabolic modeling to reveal mechanistic biomarkers with potential therapeutic relevance in pancreatic cancer."

**REVISED:** "This integrated approach combines transcriptomics data with metabolic modeling to reveal metabolic signatures and identify genes, reactions, and pathways with potential therapeutic relevance in pancreatic cancer."

**6. DISCUSSION (Multiple Revisions)**

**Location 1 (Conclusion of Multi-level Analysis Paragraph):**

**ORIGINAL:** "Overall, the integration of these three analysis levels creates a cohesive narrative of PDAC metabolism that would be impossible to achieve through any single perspective. By connecting specific gene activities to reaction fluxes and ultimately to pathway-level dysregulation, our approach bridges the gap between molecular alterations and functional metabolic consequences."

**REVISED:** "Overall, the integration of these three analysis levels creates a cohesive narrative of PDAC metabolism that would be impossible to achieve through any single perspective. By connecting specific gene activities to reaction fluxes and ultimately to pathway-level dysregulation, our approach bridges the gap between molecular alterations and functional metabolic consequences. While experimental validation is required to confirm these computational predictions, our framework provides a systematic, hypothesis-generating approach that prioritizes targets for laboratory investigation based on their discriminative power in metabolic modeling."

**Location 2 (Final Paragraph):**

**ORIGINAL:** "In conclusion, our integrated approach combining genome-scale metabolic modeling with machine learning has revealed novel insights into PDAC metabolism, identifying potential therapeutic targets and biomarkers."

**REVISED:** "In conclusion, our integrated approach combining genome-scale metabolic modeling with machine learning has revealed novel insights into PDAC metabolism, identifying potential therapeutic targets and genes whose expression could serve as biomarkers upon experimental validation."

Also, we have added the following text to the **Discussion section** to reflect the limitations of our computational approach and emphasize that our findings represent computational predictions requiring experimental validation rather than directly measurable clinical biomarkers:

First, the metabolic features identified through FBA represent computational predictions that require experimental validation before clinical application. Flux values serve as indicators of metabolic state differences but cannot be directly measured in patient samples. Future work should prioritize experimental validation of our key findings, particularly: (1) measurement of FABP, SLC27A, ACSL, and ACSBG expression levels in PDAC patient cohorts, (2) quantification of nervonic acid levels in tumors versus healthy tissue, (3) functional studies examining heparan sulfate degradation pathway activity, and (4) analysis of O-glycan profiles to confirm predicted truncation patterns.

AND

Despite these limitations, our framework provides a systematic, hypothesis-generating approach that prioritizes targets for experimental investigation based on their discriminative power in comprehensive metabolic modeling. The biological concordance of our findings with established PDAC mechanisms and experimental literature (lysosomal storage disorders, truncated O-glycans, FABP overexpression) validates our modeling approach as capable of generating meaningful, testable hypotheses about cancer metabolism and suggests that experimental follow-up studies would be fruitful.

2. FBA flux is highly related to nutrient conditions. In cancel cell, the substrates can be complex and I am not sure if the FBA can simulate the realistic cellular status. What is the objective function for cancer cell metabolism.

We thank the reviewer for raising this important methodological question. We address both concerns below:

**Objective Function:** We employed biomass maximization as the objective function for both healthy and cancer cells. This is a well-established and experimentally validated approach for modeling proliferating cells, including cancer cells. Biomass maximization assumes that cells optimize their metabolic networks to support growth and proliferation, which is particularly relevant for PDAC given its aggressive proliferative phenotype. This objective function has been extensively validated in cancer metabolism studies (references: Islam et al. 2022 - ref #3; Uhlen et al. 2017 - ref #7; Turanli et al. 2019 - ref #21) and has demonstrated good agreement with experimental flux measurements.

**Addressing Substrate Complexity and Realistic Cellular Status:** We did not use generic FBA with arbitrary nutrient conditions; instead, we employed the iMAT (integrative Metabolic Analysis Tool) algorithm, which creates context-specific metabolic models by integrating patient-specific gene expression data. This approach constrains the metabolic network based on which genes/reactions are expressed in each sample, effectively capturing the metabolic state that has already adapted to the specific microenvironmental conditions of each tumor. The transcriptomic data we integrated reflects how each cell population has adapted to its nutrient and oxygen environment—when cancer cells experience hypoxia, acidosis, or nutrient limitation, they alter gene expression accordingly (as demonstrated by our findings on glycolysis and heparan sulfate degradation pathway alterations), and the iMAT algorithm captures these adaptations by enforcing activity in highly expressed pathways while down-weighting lowly expressed ones. Furthermore, our analysis focuses on *differential* metabolism between healthy and cancerous states rather than absolute flux predictions, meaning that even if exact substrate concentrations are not perfectly captured, the systematic differences in metabolic pathway utilization between the two states remain biologically informative for understanding metabolic reprogramming in PDAC.

To address the concerns raised in this comment, we added the following part to the manuscript:

**Addition to Methods section (Genome-Scale Metabolic Modeling):**

Finally, Flux Balance Analysis (FBA) was performed to obtain reaction velocity profiles for each sample, using biomass maximization as the objective function. This is a standard approach for modeling proliferating cells that has been extensively validated in cancer metabolism research (1-3). The biomass reaction represents the production of cellular components (nucleotides, amino acids, lipids, etc.) required for cell growth and division, which is appropriate for the highly proliferative PDAC phenotype. Exchange reactions were constrained according to the default bounds in the Human1 model, representing physiological nutrient availability.

Addition to the limitations explained in the **Discussion Section**:

Second, our FBA-based metabolic modeling has inherent limitations in capturing the full complexity of the tumor microenvironment. We employed uniform exchange reaction constraints across all samples, as patient-specific metabolomic measurements (oxygen tension, nutrient uptake rates, metabolite concentrations) were not available for the TCGA dataset. While this is standard practice when only transcriptomic data are available and enables identification of gene expression-driven metabolic differences, it does not fully capture the heterogeneous tumor microenvironment, including spatial oxygen and nutrient gradients, pH variations, or temporal metabolic dynamics. Cancer cells exist in spatially and temporally heterogeneous conditions with variable oxygen, nutrient, and growth factor availability that cannot be fully represented in computational models. However, our use of the iMAT algorithm with patient-specific gene expression data partially addresses this concern by constraining metabolic models based on the actual transcriptional state of each sample, which itself reflects adaptation to microenvironmental conditions. For instance, the gene expression data integrated through iMAT captures transcriptional adaptations such as upregulation of hypoxia-responsive genes, though explicit modeling of these factors would require additional experimental data. Additionally, our use of biomass maximization as the objective function, while extensively validated for proliferating cells, may not capture all metabolic objectives of cancer cells, such as maintaining redox balance, supporting invasiveness, or surviving under stress.

AND

Third, the computational intensity of our approach may limit immediate clinical translation, suggesting the need for streamlined implementations or pre-computed signatures for clinical deployment. Future studies could integrate multi-omics data (transcriptomics, metabolomics, proteomics), implement dynamic or multi-objective FBA approaches, and develop spatially-resolved metabolic models to better represent tumor heterogeneity.

3. The model discovered the disease is associated with fatty acid transporters and acyl-CoA synthetases that drive metabolic reprogramming. Some key metabolic reactions are controlled by cell organelles (such as mitochondria and peroxisome, etc.). It is unclear to me how FBA can sensitively predict cellular responses associated with compartmentalization.

We thank the reviewer for this question, which allows us to clarify an important aspect of our modeling approach. The Human1 genome-scale metabolic model that we employed explicitly includes compartmentalization and represents metabolic reactions across multiple cellular organelles. Specifically, the Human1 model incorporates eight distinct compartments: cytosol, mitochondria, endoplasmic reticulum, Golgi apparatus, lysosomes, peroxisomes, nucleus, and extracellular space. Each metabolic reaction in the model is assigned to its appropriate compartment based on experimental evidence of enzyme localization, and transport reactions explicitly connect these compartments, allowing metabolites to move between organelles.

To address this point, we have added the following clarification to the manuscript:

**Added to Methods section (Genome-Scale Metabolic Modeling):**

The HumanGem model includes eight cellular compartments (cytosol, mitochondria, endoplasmic reticulum, Golgi apparatus, lysosomes, peroxisomes, nucleus, and extracellular space) with reactions assigned based on enzyme localization and transport reactions connecting compartments (4).

4. Augmentation of realistic synthetic healthy samples (n=4) can be a bit risky. Augmentation techniques may lead models to learn patterns that do not generalize well to actual data.

We sincerely appreciate the reviewer raising this critical concern, which touches on a fundamental challenge in pancreatic cancer research: the scarcity of healthy pancreatic tissue samples. We acknowledge that generating 140 synthetic samples from only 4 real healthy samples carries inherent risks, and we designed our methodology specifically to address these concerns through multiple validation layers. It is important to clarify why we pursued synthetic data generation rather than simply collecting additional healthy pancreatic samples from different individuals: our 4 healthy samples represent adjacent non-neoplastic tissue from the same patients who provided cancer samples, allowing for paired comparisons that control for individual biological variation. Adding healthy samples from different individuals would introduce confounding factors including race, gender, age, genetic background, and environmental exposures that would obscure cancer-specific metabolic changes. The synthetic data generation approach allowed us to expand our healthy sample size while preserving the biological context of patient-matched tissue. Unlike typical GAN applications that rely solely on statistical metrics, we implemented a comprehensive three-step biological filtration process specifically designed to identify and remove synthetic samples that do not represent realistic healthy metabolism: (1) metabolic profiling filter excluding samples with ATP, NADH, and NADPH production rates outside the range of real healthy samples, (2) glycolysis disorder index removing samples exhibiting cancer-like Warburg metabolism, and (3) gluconeogenesis assessment eliminating samples showing inappropriate activity. This stringent filtration removed some of the initially generated samples (44%), retaining only those passing all three biological criteria. Additionally, t-SNE visualization (Figure S1) demonstrates that synthetic healthy samples follow the trajectory of real healthy samples in high-dimensional gene expression space while maintaining clear separation from cancer samples, suggesting they represent interpolations within healthy biology rather than extrapolations into unrealistic space.

We have added the following text to the manuscript to clarify our rationale for using only the 4 patient-matched healthy samples rather than adding samples from different individuals:

**Added to Methods section (Data Collection and Preprocessing):**

Importantly, these 4 healthy samples represent adjacent non-neoplastic tissue from the same patients who provided cancer samples, allowing for patient-matched comparisons that control for individual biological variation (age, sex, genetic background, race, and environmental factors). This paired tissue design is crucial for isolating cancer-specific metabolic changes, as using healthy samples from different individuals would introduce confounding biological variability that could obscure disease-specific alterations.

5. lactate production in the cytosolic glycolysis pathway has been known as cancer- metabolism for many years. Can the model identify some new pathways that can be validated by recent experiments?

We thank the reviewer for this important question, which allows us to clarify a potential misunderstanding and highlight the novelty of our approach. The lactate production/glycolysis assessment was not one of our main findings but rather served as a **quality control filter** during the synthetic data validation process (Glycolysis Disorder Index in our three-step filtration). We used this well-established cancer metabolic hallmark to exclude GAN-generated synthetic samples that exhibited cancer-like metabolism, ensuring our synthetic "healthy" samples did not inappropriately display Warburg effect characteristics.

Instead, our model uncovered several novel and experimentally testable pathways through a multi-level integrated analysis that connects gene-level drivers to reaction-level flux changes and pathway-level dysregulation. For example, we identified impaired heparan sulfate degradation linked to lysosomal dysfunction in PDAC, pinpointed specific Golgi reactions responsible for truncated O-glycans, revealed coordinated regulation of nervonic acid transport implicating very long-chain fatty acid remodeling, and discovered reversed flux in bilirubin conjugation suggesting an active antioxidant strategy by cancer cells. These findings illustrate the power of our framework to move beyond known hallmarks and uncover mechanistically grounded, testable insights into PDAC metabolism.

**Reviewer #2 comments:**

1. In line 63, the manuscript states that late diagnosis and delayed treatment are major contributors to poor outcomes. Could the authors provide supporting evidence for this claim? Is it established that these factors are among the leading causes of mortality in PDAC? Clarifying this would reinforce the rationale for the study.

We thank the reviewer for highlighting this important point. The high mortality of PDAC is indeed closely tied to its late-stage diagnosis, a well-established and extensively documented challenge in the field. Approximately 80–85% of PDAC patients are diagnosed with locally advanced or metastatic disease, when curative resection is no longer possible (5) Survival rates drop sharply with disease progression. While localized PDAC has a 5-year survival rate of ~32%, this drops to just 3% for distant metastases, underscoring the urgency of early detection (6). Unfortunately, PDAC remains asymptomatic in early stages, with clinical signs such as jaundice or weight loss typically emerging only after significant progression (6). We apologize for not including proper citations earlier and have revised the manuscript as below to reflect this.

**Revised text with citations – added to introduction:**

**ORIGINAL (line 63):** Pancreatic Ductal Adenocarcinoma (PDAC) is a disease with poor prognosis and a highly aggressive form of cancer, largely due to late-stage diagnosis and limited treatment options.

**REVISED:** Pancreatic Ductal Adenocarcinoma (PDAC) is a disease with poor prognosis and a highly aggressive form of cancer, largely due to late-stage diagnosis and limited treatment options. The majority of PDAC patients (80-85%) present with locally advanced or metastatic disease at diagnosis, when curative surgical resection is no longer feasible (5). This delayed detection dramatically impacts survival, as 5-year survival rates decrease from approximately 32% for localized disease to only 3% for metastatic disease (6).

2. The manuscript mentions a lack of gene expression data from healthy samples. Is this limitation specific to the dataset used in this study? Could publicly available data from healthy individuals in other studies be incorporated? What challenges would arise from doing so?

We thank the reviewer for this thoughtful question. The limitation of having only four healthy samples reflects a deliberate design choice rather than a constraint of the dataset. These samples are patient-matched adjacent non-neoplastic tissues collected during the same resection surgery as the tumor samples. This allows us to compare cancerous and healthy tissue within the same metabolic environment and genetic background, which is critical for identifying cancer-specific alterations. Some patients in our cohort are deceased, confirming that the adjacent healthy tissue did not progress to cancer, which supports its relevance for modeling resistance mechanisms and discovering patient-specific therapeutic targets.

While publicly available healthy pancreatic data exist, they often come from patients with other diseases or from organ donors, and rarely represent truly healthy individuals, as pancreatic resection is not performed on healthy people without medical indication. Including such data would introduce bias and technical variation, and would compromise the patient-matched design. To avoid increasing dataset heterogeneity and to address class imbalance, we chose to generate synthetic samples based on the original data. This approach preserves biological context and supports our goal of developing robust cancer detection models using machine learning.

We will add clarifying text to the Data Collection and Methods sections explaining our rationale for using only patient-matched samples and the deliberate choice to generate synthetic data rather than incorporating external samples.

**Added to Data Collection and Preprocessing section:**

Additionally, adjacent healthy tissue from the same patient experiences the same systemic metabolic microenvironment as the tumor (same blood supply, circulating factors, nutrient availability) while remaining phenotypically healthy, allowing us to investigate why some tissue resists malignant transformation despite exposure to the same patient-specific conditions—a question with direct implications for identifying resistance mechanisms and patient-specific therapeutic targets. While publicly available healthy pancreatic samples from other studies exist, incorporating them would introduce significant challenges: 1. most originate from adjacent tissue of patients with other pancreatic diseases rather than truly healthy individuals, as pancreatic resection is not performed without medical indication; 2. technical batch effects from different sequencing platforms and protocols could introduce artifacts particularly problematic for metabolic modeling, which is sensitive to relative expression levels; 3. loss of the patient-matched design would prevent identification of patient-specific metabolic vulnerabilities and obscure cancer-specific signals with population-level variation. Our approach of generating synthetic samples from patient-matched tissue addresses the common class imbalance problem in cancer machine learning research while preserving the biological and technical homogeneity essential for metabolic modeling.

3. Regarding the filtering of synthetic data, why were ATP, NADH, and NADPH production levels chosen as criteria? What makes these particular metabolites critical, and why were other currency metabolites not considered?

We thank the reviewer for raising this important point about our metabolite selection criteria. We chose ATP, NADH, and NADPH because they are central indicators of cellular metabolism and directly linked to energy and biosynthesis, which are reprogrammed in tumors. Cancer cells often shift ATP production from oxidative phosphorylation to glycolysis (Warburg effect) (7). NADPH supports rapid growth and antioxidant defense, and its upregulation through the pentose phosphate pathway is well documented in cancer (8). NADH reflects the balance between glycolysis and oxidative metabolism, and changes in its levels signal metabolic reprogramming (7). These metabolites are well studied in pancreatic cancer and therefore provide reliable criteria for filtering synthetic models to represent PDAC biology.

In addition to these three, we also considered lactate production to avoid Warburg‑like patterns and gluconeogenesis activity to remove unrealistic glucose synthesis. Together, these complementary checks provided robust quality control, allowing us to exclude synthetic samples with unrealistic or cancer‑like metabolism and retain only those that reflected healthy metabolic states for comparison with cancer samples.

4. More detail is needed on how flux balance analysis was conducted following the construction of tissue-specific metabolic models using iMAT. What objective function was employed? What constraints were applied to uptake and production rates? Given the discussion of hypoxia in PDAC, was oxygen uptake treated differently across sample groups?

We used biomass maximization as the objective function across all models. This function represents the production of essential cellular components such as nucleotides, amino acids, lipids, and cofactors needed for growth and division. Biomass maximization is a well-established and widely validated approach for modeling proliferating cells, including cancer, and has been applied successfully in many cancer metabolism studies. It is particularly appropriate for PDAC, given its highly proliferative nature.

For exchange reactions, we relied on the default bounds provided in the Human1 (HumanGem) model, which reflect physiologically realistic nutrient availability under standard conditions. We did not apply sample-specific uptake or production constraints because the TCGA PDAC dataset only provides transcriptomic data and lacks metabolomic measurements such as extracellular metabolite concentrations or flux rates. We were also unable to find such data from other sources. To maintain consistency, we applied uniform exchange constraints across all samples, ensuring that the differences we observed arose from gene expression–driven network structures rather than imposed constraint variations. This is standard practice in transcriptomics-integrated metabolic modeling when metabolomic data are unavailable.

Although oxygen uptake was not set differently across groups, the integrated gene expression data already capture how cells adapt to hypoxia. In line with this, our models revealed expected metabolic changes such as increased glycolysis and altered redox metabolism. Overall, the approach successfully identified known cancer hallmarks, including changes in glycolysis and lipid metabolism, and revealed PDAC-specific features such as hypoxia-related shifts and lysosomal dysfunction. The models distinguished healthy from cancerous states with high accuracy, and the findings were consistent with experimental literature.

We acknowledge that incorporating sample-specific or condition-specific constraints, such as oxygen levels, nutrient availability, or pH, could further improve accuracy. Future work could integrate metabolomic data if it becomes available, explore dynamic or multi-condition modeling, and address spatial heterogeneity with multi-compartment tumor models. For now, given the available data and the comparative design of our study, the use of uniform exchange constraints combined with gene expression integration represents a valid and widely accepted approach that has yielded biologically meaningful insights.

We expanded the Methods section to explicitly describe the objective function and exchange constraints, which we realize were insufficiently detailed in the original submission.

**Added to Methods section (Genome-Scale Metabolic Modeling), in the FBA paragraph:**

Finally, Flux Balance Analysis (FBA) was performed to obtain reaction velocity profiles for each sample, using biomass maximization as the objective function. This is a standard approach for modeling proliferating cells that has been extensively validated in cancer metabolism research (1-3). The biomass reaction represents the production of cellular components (nucleotides, amino acids, lipids, etc.) required for cell growth and division, which is appropriate for the highly proliferative PDAC phenotype. Exchange reactions were constrained according to the default bounds in the Human1 model, representing physiological nutrient availability. These constraints were applied uniformly across all samples (healthy and cancerous), ensuring that metabolic differences identified by our models arise from gene expression-driven context-specific network structures rather than from pre-imposed constraint differences. While sample-specific metabolomic measurements (e.g., oxygen tension, nutrient uptake rates) were not available for the TCGA samples, the gene expression data integrated through iMAT captures cellular adaptation to microenvironmental conditions such as hypoxia, as evidenced by upregulation of hypoxia-responsive pathways in cancer samples.

We have added comprehensive methodological details to the Discussion section addressing the FBA modeling constraints and objective function. Specifically, we have added the following text acknowledging these limitations:

Second, our FBA-based metabolic modeling has inherent limitations in capturing the full complexity of the tumor microenvironment. We employed uniform exchange reaction constraints across all samples, as patient-specific metabolomic measurements (oxygen tension, nutrient uptake rates, metabolite concentrations) were not available for the TCGA dataset. While this is standard practice when only transcriptomic data are available and enables identification of gene expression-driven metabolic differences, it does not fully capture the heterogeneous tumor microenvironment, including spatial oxygen and nutrient gradients, pH variations, or temporal metabolic dynamics. Cancer cells exist in spatially and temporally heterogeneous conditions with variable oxygen, nutrient, and growth factor availability that cannot be fully represented in computational models. However, our use of the iMAT algorithm with patient-specific gene expression data partially addresses this concern by constraining metabolic models based on the actual transcriptional state of each sample, which itself reflects adaptation to microenvironmental conditions. For instance, the gene expression data integrated through iMAT captures transcriptional adaptations such as upregulation of hypoxia-responsive genes, though explicit modeling of these factors would require additional experimental data. Additionally, our use of biomass maximization as the objective function, while extensively validated for proliferating cells, may not capture all metabolic objectives of cancer cells, such as maintaining redox balance, supporting invasiveness, or surviving under stress.

5. Could the authors elaborate on how inherent coupling between reaction fluxes—due to the structure of the metabolic network—might influence the random forest model? For instance, reactions that are tightly coupled across all samples may exhibit similar feature importance scores. How should this affect the interpretation of the model’s outputs?

We thank the reviewer for raising this important question about how coupling between reaction fluxes might influence our random forest model. We agree that tightly coupled reactions—those linked by stoichiometric constraints or pathway structure—will often show correlated flux patterns and similar importance scores. Rather than being an artifact, this reflects genuine biological coordination. When several coupled reactions emerge as important, it indicates that entire metabolic modules, not just isolated steps, are dysregulated in cancer.

Our multi-level framework (genes → reactions → pathways) is designed to address this. Aggregating importance scores at the pathway level reduces the influence of pairwise coupling and highlights systems-level dysregulation. Consistency across genes, reactions, and pathways further validates biological relevance, while tracing reactions back to their encoding genes clarifies whether coupling reflects co-regulation, multifunctional genes, or coordinated activity across different genes.

Our results illustrate this clearly. In fatty acid metabolism, transport and activation reactions are jointly upregulated, revealing systematic reprogramming of the uptake and activation system in PDAC. In heparan sulfate degradation, sequential lysosomal reactions all show reduced activity, confirming impairment of the entire cascade. In O-glycan biosynthesis, multiple glycosyltransferase steps are disrupted together, showing that elongation as a process is affected. These examples demonstrate that coupled reactions provide meaningful insight into pathway-level dysfunction.

We interpret similar importance scores among coupled reactions as evidence of coordinated metabolic programs, robust biomarkers, and multiple potential intervention points. Coupling also provides internal consistency checks, since changes in one reaction should be mirrored in its partners. We acknowledge challenges such as possible overestimation of pathway importance, difficulty in identifying the true driver within a coupled set, and redundancy in importance scores. To mitigate this, we normalize pathway-level scores, trace findings back to gene-level drivers, and use clustering to visualize reaction groupings.

While assigning causality within tightly coupled sets remains difficult, these limitations stem from the structure of metabolic networks rather than flaws in our approach. Our multi-level analysis helps navigate these complexities, and we will add clarifying text to the manuscript to explain how coupled reactions are interpreted and how our framework contextualizes coordinated metabolic changes.

We will add clarifying text to the Results or Discussion explaining how coupled reactions are interpreted in our framework and how the multi-level analysis provides context for understanding coordinated metabolic changes.

**Added to Discussion Section:**

It is important to note that reactions within metabolic pathways are often stoichiometrically or functionally coupled, meaning they must carry correlated fluxes. When our random forest model identifies multiple coupled reactions as important features, this reflects biologically meaningful coordination rather than statistical redundancy. For example, our identification of both fatty acid transport reactions (encoded by FABPs and SLC27As) and subsequent activation reactions (encoded by ACSLs and ACSBGs) as highly important features reveals that the entire fatty acid uptake and processing system is coordinately upregulated in PDAC (Figure 8C). Our multi-level analysis framework specifically addresses reaction coupling by: (1) aggregating reaction importances to the pathway level, which dampens the influence of individual coupled pairs while highlighting overall pathway dysregulation, (2) tracing coupled reactions back to their encoding genes to reveal mechanistic basis, and (3) using hierarchical clustering (Figure 3A) to explicitly visualize reaction groupings. This approach allows us to interpret coupled reactions as indicators of coordinated metabolic reprogramming—identifying entire metabolic modules rather than isolated reactions as dysregulated in cancer, which provides more actionable biological insights.

6. For Figure 4A, would a box plot or a probability density plot for each flux provide a clearer and more intuitive visualization of the results?

We thank the reviewer for this excellent suggestion. We agree that probability density plots provide a clearer and more intuitive visualization of the flux distributions. We have revised Figure 4A to replace the parallel coordinate plot with Kernel Density Estimation (KDE) probability density plots for each of the three heparan sulfate degradation reactions and the revised figure 4 is included in the attachments.

Revised Figure 4A caption in the manuscript:

**(A) Distribution of Heparan Sulfate Degradation Reaction Fluxes in Lysosomes between Healthy and Cancerous States.** Probability density plots (Kernel Density Estimation) comparing the flux distributions of three key lysosomal degradation reactions (MAR07238, MAR07241, MAR07248) processing specific heparan sulfate degradation products (HS-Deg 13, HS-Deg 16, and HS-Deg 23) between healthy (blue) and cancerous (red) samples (n=144 each). Vertical dashed lines indicate mean flux values for each condition. The consistently lower flux values and leftward shift of cancer distributions across all three reactions demonstrate reduced activity of the Heparan Sulfate Degradation pathway in PDAC, aligning with the observed decrease in HSPG exchange at the cell boundary.

Also, we made the following change to the explanation of this figure in section 3.5.1 of the manuscript:

As shown in **Fig 4A**, probability density distributions comparing healthy and cancerous states reveal a consistent trend across all three key lysosomal degradation reactions (MAR07238, MAR07241, MAR07248). The overlaid density curves show healthy samples in blue and cancerous samples in red, with vertical dashed lines indicating mean flux values for each condition. Notably, all three reactions exhibited substantially lower activity in cancerous cells, as evidenced by the pronounced leftward shift of the cancer distribution (red) compared to the healthy distribution (blue) and the lower mean flux values (red dashed lines vs. blue dashed lines).

7. Do the observed trends in reaction fluxes and pathway differences hold when analyzing only the real (non-synthetic) samples? While statistical significance may be limited due to the small number of healthy samples, does the real data support the same patterns?

We thank the reviewer for this critical validation question. The comparison of flux patterns between real healthy samples (n=4), synthetic healthy samples (n=140), and cancer samples (n=144) demonstrates that the patterns are consistent, with cancer samples showing the same directional changes relative to both real and synthetic healthy samples. This consistency is evident in our top-ranked findings:

**Nervonic Acid Transport (MAR00336 - Top-ranked reaction):**

- Real-healthy mean flux: 0.15
- Synthetic-healthy mean flux: 0.14
- Cancer mean flux: 6.54

Cancer samples show 44-fold higher activity compared to real healthy samples and 46-fold higher compared to synthetic healthy samples, demonstrating dramatic and consistent upregulation regardless of whether we compare to real or synthetic healthy tissue.

**Heparan Sulfate Degradation (Top-ranked pathway):**

For the three key lysosomal degradation reactions:

**MAR07238:**

- Real-healthy: 23.3, Synthetic-healthy: 19.4, Cancer: 6.7
- Cancer shows 3.5-fold reduction vs real-healthy and 2.9-fold reduction vs synthetic-healthy

**MAR07241:**

- Real-healthy: 23.3, Synthetic-healthy: 16.3, Cancer: 3.4
- Cancer shows 6.9-fold reduction vs real-healthy and 4.8-fold reduction vs synthetic-healthy

**MAR07248:**

- Real-healthy: 23.3, Synthetic-healthy: 16.3, Cancer: 3.4
- Cancer shows 6.9-fold reduction vs real-healthy and 4.8-fold reduction vs synthetic-healthy

Cancer samples consistently show the same directional changes compared to both real and synthetic healthy samples, with large fold‑changes that are robust across groups. The synthetic healthy data closely matches the real healthy distribution, and both differ sharply from cancer, confirming that the observed patterns are genuine PDAC‑associated alterations rather than artifacts. Although the number of real healthy samples is small, their agreement with synthetic data strengthens confidence in the findings, and we will add a supplementary table comparing mean flux values across groups to make this validation clear.

Added to the supplementary files:

**Supplementary Table S1: Validation of Key Metabolic Findings in Real Healthy Samples**

| **Reaction/Pathway** | **Real-Healthy (n=4) Mean Flux** | **Synthetic-Healthy (n=140) Mean Flux** | **Cancer (n=144) Mean Flux** | **Fold-Change (Cancer vs Real-Healthy)** |
| --- | --- | --- | --- | --- |
| **MAR00336** (Nervonic acid transport) | 0.15 | 0.14 | 6.54 | +44.0× |
| **Heparan Sulfate Degradation** | | | | |
| MAR07238 | 23.3 | 19.4 | 6.7 | -3.5× |
| MAR07241 | 23.3 | 16.3 | 3.4 | -6.9× |
| MAR07248 | 23.3 | 16.3 | 3.4 | -6.9× |

Table shows that cancer-associated metabolic patterns (increased nervonic acid transport, decreased heparan sulfate degradation) are consistent when comparing cancer samples to either real or synthetic healthy samples, validating that findings are not artifacts of synthetic data generation.

Also, the following text was added to section 3.5.1 in the manuscript:

Importantly, these patterns are consistent when comparing cancer samples to the 4 real healthy samples directly, not just to synthetic samples (**Table S1**), with fold-changes of similar magnitude, confirming that our findings are not artifacts of synthetic data generation.

In addition, the following text was added to section 3.6 which is related to the most important reaction (nervonic acid transport):

This elevated transportation of nervonic acid in PDAC likely reflects a significant metabolic adaptation in this disease that is validated in real healthy samples (**Table S1**).

8. The study presents a method for analyzing gene expression data across conditions with unequal sample sizes by incorporating validated synthetic data generated via GANs. It would be helpful for the authors to discuss the risks, limitations, and necessary precautions when interpreting results derived from synthetic data.

We thank the reviewer for this important question, which allows us to provide a comprehensive discussion of the appropriate use and interpretation of synthetic data in computational biology. This is a critical consideration for the broader application of such methods. We added a dedicated subsection to the Discussion section as below to address these risks, limitations, and precautions explicitly. This will help readers contextualize our findings appropriately and understand the scope and limitations of conclusions that can be drawn.

**Added to the Discussion Section:**

Our use of GAN-generated synthetic healthy samples addresses a critical challenge in cancer research—severe class imbalance in patient-matched study designs—but requires careful interpretation. Synthetic samples generated from only 4 real samples cannot capture the full biological diversity of healthy pancreatic tissue and are not statistically independent observations. While our rigorous three-step biological filtration (rejecting 44% of generated samples) ensures metabolic validity for tested characteristics, it cannot validate all biological constraints or gene regulatory relationships. Importantly, synthetic data may amplify idiosyncratic features of the original samples rather than universal healthy characteristics, and traditional statistical tests assuming independent samples may produce overconfident estimates.

We implemented several safeguards to mitigate these risks: multiple orthogonal biological validation criteria, t-SNE visualization confirming appropriate high-dimensional clustering, multi-level consistency checks across genes-reactions-pathways, and systematic comparison with established cancer biology literature. The biological concordance of our major findings (lysosomal dysfunction aligning with recent genetic studies, O-glycan patterns matching known cancer glycosylation, lipid metabolism changes consistent with experimental validations) provides external validation that synthetic data did not introduce spurious patterns. Nevertheless, our results should be interpreted as hypothesis-generating computational predictions rather than definitive findings. The identified metabolic signatures require validation in independent cohorts of real samples and experimental confirmation through functional studies.

We emphasize that synthetic data cannot replace real data collection. Our approach represents a pragmatic solution for exploratory analysis and target prioritization when severe data scarcity exists, but the identified metabolic features must be validated experimentally before clinical application. Future studies should prioritize: (1) expanding real healthy pancreatic tissue biobanks, (2) multi-center collaborations to aggregate larger patient-matched cohorts, (3) experimental validation of predicted metabolic alterations through lipidomics, metabolomics, and functional assays, and (4) replication of findings in independent real datasets. The value of our framework lies not in providing definitive answers but in systematically prioritizing targets for such validation studies within the complex landscape of cancer metabolism.

9. Line 152: The phrase should be revised to “deal with the class imbalance issue.”

We thank the reviewer for catching this grammatical error. We have corrected line 152 as suggested.

**Original:** "...proposed an enhanced generative adversarial network (E-GAN) to deal the class imbalance issue..."

**Revised:** "...proposed an enhanced generative adversarial network (E-GAN) to deal with the class imbalance issue..."

1.	Islam MM, Goertzen A, Singh PK, Saha R. Exploring the metabolic landscape of pancreatic ductal adenocarcinoma cells using genome-scale metabolic modeling. Iscience. 2022;25(6).

2.	Turanli B, Zhang C, Kim W, Benfeitas R, Uhlen M, Arga KY, et al. Discovery of therapeutic agents for prostate cancer using genome-scale metabolic modeling and drug repositioning. EBioMedicine. 2019;42:386-96.

3.	Uhlen M, Zhang C, Lee S, Sjöstedt E, Fagerberg L, Bidkhori G, et al. A pathology atlas of the human cancer transcriptome. Science. 2017;357(6352):eaan2507.

4.	Robinson JL, Kocabaş P, Wang H, Cholley P-E, Cook D, Nilsson A, et al. An atlas of human metabolism. Science signaling. 2020;13(624):eaaz1482.

5.	Mizrahi JD, Surana R, Valle JW, Shroff RT. Pancreatic cancer. The Lancet. 2020;395(10242):2008-20.

6.	Rawla P, Sunkara T, Gaduputi V. Epidemiology of pancreatic cancer: global trends, etiology and risk factors. World journal of oncology. 2019;10(1):10.

7.	Heiden MGV, Cantley LC, Thompson CB. Understanding the Warburg effect: the metabolic requirements of cell proliferation. science. 2009;324(5930):1029-33.

8.	Patra KC, Hay N. The pentose phosphate pathway and cancer. Trends in biochemical sciences. 2014;39(8):347-54.
